# Editorial: Advanced insights into plant rhizosphere functionality from the perspective of declining soil fertility status in the era of climate change

**DOI:** 10.3389/fpls.2023.1338575

**Published:** 2023-11-23

**Authors:** Faisal Nadeem, Xingxing Liu

**Affiliations:** ^1^ Department of Soil Science, University of the Punjab, Lahore, Pakistan; ^2^ State Key Laboratory of Plant Physiology and Biochemistry, College of Natural Resources and Environmental Science, Zhejiang University, Hangzhou, China

**Keywords:** soil pH, struvite, organic amendments, salt-affected soils, temperature-dependent N application

Rhizosphere functionality influences the strategic nutrient acquisition by various plant species. The intensification of agricultural practices, ensuring global food security, needs re-evaluation, focusing on the challenges faced by agricultural soils in the era of climate change. In this regard, five original studies were attracted by this Research Topic, the key findings of which will be summarized in this editorial.

Considering the rhizosphere microbial community, the root exudations cause alterations in their composition through interactions with soil substances (Tan et al., 2022). Ye et al. reported the growth, yield, and quality of tea crop regulated by the changes in microorganisms, related metabolites, and physicochemical properties of rhizosphere soils under different pH conditions. The increased rhizosphere soil pH enhanced organic matter content, cation exchange capacity and microbial growth except the fungi. These enhancements were further positively related to the total metabolite contents of tea rhizosphere soil. These findings, reporting the metabolomics analysis of characteristically functional metabolites, suggested that the increase in tea rhizosphere soil pH could be an effective strategy to improve the diversity and number of soil microorganisms and enhance carbon (C)/nitrogen (N) cycling for the growth promotion of tea crop. Talking about rhizosphere dynamics, the sorption and precipitation-dependent immobilization of phosphorus (P) in soils significantly reduces its availability to plant roots (Rech et al., 2018). Struvite (MgNH_4_PO_4_·6H_2_O), a slow release fertilizer (Talboys et al., 2016), has the potential to overcome P constraints in such soils (Rech et al., 2018) along with its additional benefits of nitrogen (N) and magnesium (Mg) supply for plant development (Kataki et al., 2016). Valle et al. demonstrated the benefits of polysulfide matrix (PS) and dispersed struvite (St) applications in soybean cultivation system. The enhancements in total root length (1,942-4,291 cm) of soybean plants under St/PS composites, compared to triple superphosphate/Ammonium sulfate (982 cm), explained the overwhelming impact of St/PS. Furthermore, the St/PS composites showed their slow release potential by maintaining P uptake efficiency at par with all other fertilized treatments (11–14%); yet achieving 22% sulfur uptake efficiency compared to only 8% from TSP/AS applications.

In addition to P, carbon (C) and nitrogen (N) are also known as the limiting factors influencing soil productivity, plant growth and development, and metabolism (Gu et al., 2019). They have a direct influence on photosynthesis, nutrient uptake and fruit development (Sivanandhan et al., 2015). As N nutrition always shifts the plant C pool (Zhou et al., 2022) hence, it becomes necessary to understand further the processes of their fixation, allocation and transfer within soil and plant system (Wang et al., 2020). Zhang et al. revealed the impact of temperature-dependent N application on soil C and N accumulation along with the bacterial community diversity in the rhizosphere soil under *Malus sieversii* cultivation. The increases in plant ^13^C (P-Atom^13^C) abundance, plant ^15^N (P-Con^15^N) abundance and soil ^15^N (S-Atom^15^N) abundance together with soil urease, protease and glutaminase activities under nitrogen application at the same temperature level indicated rhizosphere soil enrichments. N application (N_1_) also improved bacterial community diversity and richness indices. At low temperature, applying N uplifted the relative abundances of *Actinobacteria*, *Rhodopseudomonas* and *Bradyrhizobium*. Redundancy analysis (RDA) showed that plant ^13^C (P-Atom^13^C) abundance and plant ^15^N (P-Con^15^N) abundance were pivotal factors that benefited the soil bacterial community composition under low temperature. Thus, N nutrition can alleviate the low temperature stress in the soil through betterments of soil bacterial community compositions for the uptakes of carbon and nitrogen in *Malus sieversii* plants.

Salt-affected soils are the problem of arid and semi-arid regions worldwide. 25% of the farmland (99.13 million hectares) of China accounts for salt-affected soils (Zhao and Li, 1999). With respect to cereal crops, alfalfa presents more sensitivity to salt stress (Isayenkov, 2012). The continuous evapo-transpiration during the spring season elevates the salinization of the top soil layer, which poses serious hazards for alfalfa emergence and growth (Guan et al., 2019). The negative impact of salinity on alfalfa is probably imposed through the decline in its N nutrition constraints, as reported in *Glycine max* (Ghassemi-Golezani et al., 2010). Wan et al. investigated the role of N supply in improving alfalfa yield and quality under salt stress conditions. They reported improvements in shoot dry weight (40%–45%), root dry weight (23%–29%) and shoot N content (10%–28%) through increased N supply in alfalfa. These enhancements resulted from improved fixation of N derived from the atmosphere (47%-60%) under salt stress. The offset of the negative effects of salt stress on alfalfa growth and N fixation through improvements in N nutrition status suggested the importance of optimal N fertilization in salt-affected soils.

Excessive synthetic fertilizers application compromises soil health and pollutes the environment (Cui et al., 2020). The orchard ecosystem services can be improved by using various organic matter (OM) based amendments (Montanaro et al., 2017). Application of OM can conserve resources such as water and nutrients (Novara et al., 2021) and achieve broader sustainable development goals through carbon sequestration (Lal et al., 2021). Composting, as an OM amendment, can potentially improve yield in apple (Moran and Schupp, 2005) and peach (Montanaro et al., 2012). Lawrence and Melgar studied the replacement of synthetic fertilizer with compost for the improvement of nutrient and water status during the first four years of peach orchard development in subtropical climate. Composting enhanced soil OM, P and sodium (Na) in 15 cm soil depth of replant location as compared to the virgin location. During the growing season, it also proved to improve soil moisture and, subsequently, increased fruit yield in the virgin location as well. Thus, the incorporation of food waste compost at a 2x rate can replace synthetic fertilizers and potentially increase peach growth, at least during orchard establishment.

These findings provide new insights for future investigations involving pH-dependent rhizosphere modifications, struvite as a solution to P constraints, rhizosphere temperature-dependent C and N accumulations, N management in salt-stressed legumes and composting as a replacement for chemical fertilizers.

## Author contributions

FN: Conceptualization, Writing – original draft. XL: Writing – review & editing.
